# Coping strategies for stress used by adolescent girls in Riyadh, Kingdom of Saudi Arabia

**DOI:** 10.12669/pjms.305.5014

**Published:** 2014

**Authors:** Hafsa Raheel

**Affiliations:** 1 Hafsa Raheel, Assistant Professor, Department of Family & Community Medicine, King Khalid Hospital & King Saud University, Riyadh, Kingdom of Saudi Arabia.

**Keywords:** Adolescents, Coping strategies, Stress, Riyadh, Saudi Arabia

## Abstract

***Objectives: ***Secondary school girls, ages 15 – 19 yrs were surveyed to find out the coping strategies they used when stressed. Adolescents, who are affected with stress and depression early in life, suffer from depression throughout their lives especially if they are utilizing improper ways to cope with it.

***Methods:*** A cross sectional school based survey among 1028 adolescent girls was conducted among the secondary schools in Riyadh city,Kingdom of Saudi Arabia.

***Results: ***About 25% stated that they cry, 19% listen to music, 15% start eating a lot, 12% sit alone/isolate themselves, 11% pray/read the Quran, 10% get into a verbal argument or a fight. Only a few, 3% exercise, and 2% stated that they find someone to discuss and talk to.

***Conclusion:*** Majority of the adolescent girls in our survey, rely on emotion related coping mechanisms rather than problem solving mechanisms. This can cause long term implications in these adolescents as there is an increased probability to develop depression later on in life. Policy makers need to implement strategies for early identification of stress and depression. Talking to friends and family can serve as an effective way to cope with stress.

## INTRODUCTION

In 2011, the State of World Children, UNICEF reported that around 20 per cent of the world’s adolescents have a mental health or behavioral problem. Depression is said to be single largest contributor to the global burden of disease among people aged 15–19 years.^1^ It has been reported by Costello in 2006, that in UK, at any given point in time approximately 4 – 6% of adolescents ages 13 – 17 are clinically depressed.^2^

Research studies report that those adolescents who are affected with depression early in life, often suffer from depression throughout their lives and in many cases, early onset of depression predicts severe depression later during adulthood.^3^^,^^4^ Other than an increase in suicidal tendency, youth who are depressed are at a higher risk of mental disorders such as anxiety, conduct disorders, and substance abuse.^3^^,^^4^ Although depression in adolescents can be related to multiple factors, biological, cognitive and environmental, Terje A et al. proposed and researched a stress-depression model, which states that the environmental or social stressors are likely to be moderated by an individual’s coping styles.^5^

Lazarus defined coping as a process in which cognitive or behavioral efforts are made to manage specific internal and /or external sources of psychological stress.^6^ Coping has been broadly categorized into problem-focused and emotion-focused coping. In problem –focused coping, the individual works to change the circumstances causing stress, such as if the main stressors is the fear of failing an exam, problem solving strategies could be; putting extra efforts towards learning that subject or working extra hard to gain marks in other ways as class work or home works. In emotion-focused coping the person works and tries to minimize the stress caused by the stresses such as; relaxation techniques, crying, yelling etc.^7^^,^^8^

Studies in the Middle East and Saudi Arabia have reported depression among adolescents and its correlates.^9^^,^^10^ None of these studies reported the coping strategies these adolescents are using to overcome their depression. Therefore, identifying the gap in literature and the need to explore what mechanisms these adolescents are using to cope with stress, we designed this study with an aim to advise on better ways to cope with stress and depression, resulting in good mental health of the adolescents.

## METHODS

***Design: ***A cross sectional, school based survey was conducted among the secondary schools in Riyadh city. A sample of 1028 adolescent girls aged 15-19 years was enrolled from secondary schools. The study was designed to investigate the coping strategies these young adolescent girls are adopting to cope with daily stressors. 

***Setting & Study population: ***Riyadh is the capital of the Kingdom of Saudi Arabia and has a population of about 7 million people. It is the center of education for the country with one of the best educational opportunities available. The educational system of the country has separate school and Universities both for males and females, from primary to higher secondary and University level.

For the sake of a representative sample, Riyadh was divided into five clusters, south, east, north, west, and central. A list of secondary schools (having educational classes till 12^th^ grade) was obtained from the Ministry of Higher Education. We tried to enroll at least one private and one public school from each cluster. However, due to security reason, and constraints that data collection was done by females only, the southern region was excluded from the study. The schools selected within each cluster were approached after obtaining approval from the ministry of higher education and school administration.

***Inclusion and exclusion criteria: ***Girls studying in grades 10-12, irrespective of the language and nationality were enrolled. Those who were suffering from any mental conditions like, schizophrenia and phobia were excluded from the survey. Also those previously diagnosed as depressed and on anti-depressants were not enrolled.

***Enrolment method: ***Within each school, a list of students in grades 10 – 12 was obtained and within each class every alternate student according to the class enrolment sheet was offered to participate in the survey. Participation was voluntary.

***Questionnaire:***

***Details of the tool used: ***A structured questionnaire was developed to be filled in by the respondents.

*The first part* consisted of variables related to the socio-demographic profile of the participants. Age, nationality, school grade, type of school, marital status, number of children if married, accommodation, respondents’ living structure, parents education, family income, previous history of depression, academic scores, and history of medication, specially usage of antidepressants. 

The final section consisted of a list of coping mechanisms adopted by adolescents when in stress, mentioned in literature. A simple definition was used to define *Stress *in our survey as; “a state of mental or emotional strain or tension”. The respondents were asked which of these strategies they used if they were stressed, and to mention any other way that they dealt with stress, and was not mentioned in the list.

The questionnaire was constructed based on evidence from literature and consultations provided from field experts. It was pretested among 100 adolescent female students. The school where the pretesting was done was excluded from the actual survey. After the pretesting, required changes were made in the questionnaire. Informed consent was obtained and a code was assigned to each questionnaire, which was entered in the data system, this was to ensure anonymity of the respondents. Ethical approval was obtained from the University ethical approval board. 

## RESULTS

Interviews were conducted with 1028 adolescent girls. Table-I shows the sociodemographic data of the survey participants. 

A majority of the participants were of 16 – 17 yrs of age. Only a few were non Saudi, 2/3^rd^were studying at a private school. A large lived in independent villas. Ninety one percent fathers of girls were educated at bachelor or higher levels. On the other hand 67% of mothers had bachelor or higher degree.

At the last exam, Seven hundred and thirty five of these students (72%) scored excellent grades (100-90%), 229 (22%) very good (89 -80%), 53 (5%) good (79 – 70%), and 7 (0.5%) fair grades (69 – 60%).

Coping strategies adopted by the respondents are shown in Table-II. About 139 (25%) stated that they cry, 104 (19%) listen to music, 78 (15%) start eating a lot, 65 (12%) sit alone/isolate themselves, 63 (11%) pray/read the Quran, 57 (10%) get into a verbal argument or a fight. About 15 (3%) exercise, 12 (2%) stated that they find someone to discuss.

**Table-I:** Table showing the basic sociodemographic profile of the study participants

***Variable***	***n =1028 (%)***
**Age of respondents (yrs)**	
15 16 17 18 19	111 (11) 352 (34) 369(36) 183 (18) 14(1)
**Nationality**	
Saudi Non- Saudi	941 (91.5) 87(8.5)
**School Grade**	
10^th^ 11^th^ 12^th^	317 (31) 340 (33) 371 (36)
	
**School type**	
Private Public	750 (73) 278 (27)
**Type of accommodation living in**	
Villa Apartment One floor flat Others	903 (88) 82 (8) 31 (3) 12 (1)
**Order of respondent among sibling**	
Eldest Middle Youngest	259 (25) 607 (59) 162 (16)
**Father’s education level **	
Less than secondary (< 10^th^ grade) Secondary (11 -12) Bachelor’s degree Master’s degree Doctorate Others	16 (2) 75 (7) 366 (36) 154 (15) 247 (24) 170 (17)
**Fathers Occupation**	
Doctor Teacher Engineer Business Military Banker Unemployed Others	98 (10) 85 (8) 156 (15) 248 (24) 81 (8) 37 (4) 13 (1) 310 (30)
**Mother’s education level **	
Less than secondary (< 10^th^ grade) Secondary (11 -12) Bachelor’s degree Master’s degree Doctorate Others I don’t know	79 (8) 252 (25) 467 (45) 68 (7) 45 (4) 21 (2) 96 (9)
**Mother’s Occupation**	
Doctor Teacher Housewife Business Banker Others	28 (3) 263 (26) 569 (55) 31 (3) 18 (2) 119 (12)
**Academic scoring **	
Excellent (100 - 90%) Very good (89 – 80%) Good (79 – 70%) Fair (69 – 60%) Repeater (< 60%)	735 (72) 229 (22) 53 (5) 7 (0.5) 4 (0.5)

**Table-II:** Table showing the various coping strategies adopted by the adolescent female study participants

***Coping strategy***	***N= 549 (%)***
Cries	139 (25)
Listen to music	104 (19)
Starts eating a lot	78 (15)
Sits alone/isolation	65 (12)
Prays /reads Quran	63 (11)
Gets in a verbal argument/ starts a fight	57 (10)
Exercises	15 (3)
Gets into a physical fight	12 (2)
Finds someone to talk to	6 (1)
Smokes	3
Others	11 (2)

The responses were noted as multiple responses.

## DISCUSSION

People suffer from stress for various reasons but stress is not that harmful; what is harmful is the incorrect coping strategies that has a negative impact on the mental health of adolescents and caries its effects later on in adulthood.^11^^,^^12^

Our study highlighted that majority of the adolescent’s girls in our survey, rely on emotion related strategies rather than problem solving strategies. They cope in isolation, as they tend to use emotional strategies rather than addressing and trying to resolve the stressors. This can cause long term implications as there is an increased probability to develop depression later on in life. McCrae and Carver state that the tendency to react aggressively at times of stress is a reflection of hopelessness, which in turn may result in depression and aggravated stress. Such coping styles may lead to more conflicts and rejection towards peers, teachers and family.^13^ Emotion focused coping strategies are also found to be associated with mental health problems such as anxiety and depression.^13^^,^^14^ In our study, only a small percentage (1%) of the respondents were using problem solving strategies, such as talking to someone. Coping strategies have been found to have an impact on life. Problem-focused coping strategies, such as actively discussing the issue, facing the stressors and active ways to overcome stress have shown to decrease depression among adolescents and later on in adulthood.^8^^,^^12^ Majority of the respondents were relying on avoidance strategies, such as sitting alone/isolating themselves, and crying. While others fight low mood and stress aggressively (emotion focused) by getting into arguments or fight with others. These findings are similar to findings of a study conducted by Liu et al, in which only a few of their respondents utilized problem focused strategies and majority were utilizing maladaptive strategies.^15^ The findings supported that adolescents need to be trained in overcoming maladaptive strategies in order to avoid depression and suicidal tendency in the future.

Around 11% of our participants stated that they read The Quran or prayed when stressed or depressed. Coping stress by ways of religious activities has been reported by females more than males.^16^ Religious individuals tend to place a greater value on the act of forgiveness and subjects report higher levels of forgiveness. Devon M and colleagues conducted a longitudinal study among college students in the US Universities, and reported that religiosity and spirituality played a protective role against development of depression.^17^ In another meta-analytic review published in 2012, authors found direct correlation between religiosity and spirituality and outcome measures; reduce risk behavior and depression, and increased self-esteem, and well-being.^18^

A surprising finding in our study is that none of the adolescents relied on family support or peers support. Studies have reported that adolescents find great relief when they talk to their parents and friends.^19^ One reason could be that these girls may not be finding quality time together with each other, as female mobility is limited within the Saudi culture. Females have to rely on the males in the house, either fathers or brothers and drivers for movement and socialization outside the homes, which can be a reason for limited interaction and socialization with their peers, leading to limited discussions and building of a good and open environment for exchanging personal behaviors and experiences and building trusting friendships among them.

We would like to make some suggestions based on our findings; first and foremost, we need to create awareness among families, friends, and teachers regarding the fact that about 30% of adolescents are stressed to such an extent that it makes them cry. Second step is to help these adolescents know how they can cope better with the stressors for a better and healthier mental health. There is a need to identify and build in mechanisms for early and timely diagnosis of stress among adolescents and provide support to adolescents to adopt problem solving coping strategies rather than emotion focused, to combat stress, in an attempt to decrease the development of depression and anxiety latter on in adulthood. An intervention that has been proved ‘effective in this regard, is through schools. Councilors or school teachers can be trained in identifying the early signs of stress and depression among their pupils and provide early interventions.^5^ Adolescents need to be trained to focus on problem-solving strategies and avoid maladaptive coping mechanisms when exposed to stressful situations. They need to be taught how to effectively resolve their daily stressors in order to prevent depression.^20^

Peer groups can be formulated and trained at the school level who can help their colleagues in coping with stress. Also parents can be educated and guided to be supportive and understanding of the common stressors among this age group and how to help their youngsters at the time of stress. The effectiveness of communication with parents, especially mothers can play a positive role in supporting the girl adolescents. Talking about their daily problems, and sharing of experiences in ways to handle stress, needs to be emphasized. Role of media cannot be overlooked. Adolescents are seen to be fascinated and influenced by famous leaders and icons, which can be involved in promoting mental health and its concepts among the youth. They can help in advising adolescents with regard to better ways to address their stress. Lastly but not least religious activities need to be promoted as a source and way to combat stress and depression, in both educational and home environment. We would recommend further research on interventions in which religious activities have been employed and its effects on controlling stress among adolescents. All these need national policies to be formulated targeted towards promoting healthy adolescent mental health.

*Limitations:* While the study has highlighted the coping strategies and provided important information for policy and program development. We can mention some limitations to the generalizability of our findings. Most important being that the study has been conducted among educated adolescent girls from city and for that reason the findings may not be the same when rural adolescents, or adolescent boys are studied. The cultural and social factors in smaller cities and towns and rural areas, and different socioeconomic strata are much different from those in larger cosmopolitan cities and higher socioeconomic strata.
